# Analysis of Biomarkers for Congenital Heart Disease Based on Maternal Amniotic Fluid Metabolomics

**DOI:** 10.3389/fcvm.2021.671191

**Published:** 2021-06-07

**Authors:** Yahong Li, Yun Sun, Lan Yang, Mingtao Huang, Xiaojuan Zhang, Xin Wang, Xianwei Guan, Peiying Yang, Yan Wang, Lulu Meng, Ran Zhou, Xiaoyan Zhou, Chunyu Luo, Ping Hu, Tao Jiang, Zhengfeng Xu

**Affiliations:** ^1^Center of Prenatal Diagnosis, Women's Hospital of Nanjing Medical University, Nanjing Maternity and Child Health Care Hospital, Nanjing, China; ^2^Department of Prenatal Diagnosis, Wuxi Maternity and Child Health Hospital Affiliated to Nanjing Medical University, Wuxi, China; ^3^Department of Obstetrics, The Affiliated Huaian No, 1 People's Hospital of Nanjing Medical University, Huaian, China

**Keywords:** congenital heart disease, metabolomics, pregnancy, biomarker, amniotic fluid

## Abstract

Congenital heart disease (CHD) is the most common birth defect. The prenatal diagnosis of fetal CHD is completely dependent on ultrasound testing, but only ~40% of CHD can be detected. The purpose of this study is to find good biomarkers in amniotic fluid (AF) to detect CHD in the second trimester, so as to better manage this group of people and reduce the harm of CHD to the fetus. Metabolites analysis were performed in two separate sets. The discovery set consisted of 18 CHD fetal maternal AF samples and 35 control samples, and the validation set consisted of 53 CHD fetal maternal AF samples and 114 control samples. Untargeted metabolite profiles were analyzed by gas chromatography/time-of-flight-mass spectrometry (GC-TOF/MS). Orthogonal partial least square discrimination analysis (OPLS-DA) demonstrated that CHD and control samples had significantly different metabolic profiles. Two metabolites, uric acid and proline, were significantly elevated in CHD and verified in two data sets. Uric acid was associated with CHD [odds ratio (OR): 7.69 (95% CI: 1.18–50.13) in the discovery set and 3.24 (95% CI:1.62–6.48) in the validation set]. Additionally, uric acid showed moderate predictive power; the area under curve (AUC) was 0.890 in the discovery set and 0.741 in the validation set. The sensitivity and specificity of uric acid to detect CHD was, respectively, 94.4 and 74.3% in the discovery set and 67.9 and 71.9% in the validation set. The identification of uric acid as a biomarker for CHD has the potential to stimulate research on the pathological mechanism of CHD and the development of a diagnostic test for clinical applications.

## Introduction

Congenital heart disease (CHD) is the most common birth defect that is defined as a cardiovascular malformation caused by abnormal vascular dysplasia during fetal development (1). CHD affects about 1% of live births and has become the leading cause of childhood mortality (2, 3). Despite incomplete knowledge on the onset and development of CHD, recent findings indicate that intrauterine infection, environmental factors, chromosomal abnormalities, gene mutations and epigenetics are involved in this disease (4–7). CHD phenotypes are diverse, involving pulmonary stenosis, aortic stenosis, atrial septal defect, aortic transposition, right ventricular double outlet, ventricular septal defect, patent ductus arteriosus, tetralogy of Fallot, and other types of defects (8). The impact of CHD on patients is different due to severity, and severe CHD may affect the survival and quality of life of patients (9, 10). The prenatal diagnosis of fetal CHD is completely dependent on ultrasound testing, but the accuracy is affected by various factors, such as tester expertise, equipment quality, fetal position, and maternal obesity. CHD type is another important factor affecting detection, and most CHD is not discovered until after the birth (11). Only ~40% of CHD can be detected by prenatal ultrasound (12). If as many CHD patients as possible can be detected early in pregnancy, this will be helpful for this group of people to conduct prenatal consultation, receive timely obstetric management, proceed postpartum diagnosis and treatment, which may improve the treatment effect and quality of life. However, due to the low detection rate, the exploration of CHD-related biomarkers is still urgently needed.

The concept of metabolomics was firstly proposed by Nicholson et al. (13). Metabolomics is widely used in the field of life science to study endogenous metabolites of the whole body and their changes (14). Metabolomics provides the methods for the identification of the changes in the metabolite profiles of biological fluid to support early biomarker discovery, disease diagnosis and treatment (15). Several metabolomics studies have focused on fetal CHD, and we summarized these studies as shown in Table 1. In these studies, the types of samples used were maternal serum, maternal urine and amniotic fluid (AF), and the methods used were direct injection/liquid chromatography and tandem mass spectrometry (DI/LC-MS/MS), tandem mass spectrometry (MS/MS), gas chromatography-mass spectrometry (GC-MS) and nuclear magnetic resonance (NMR) (16–20). However, studies of fetal CHD based on amniotic fluid metabolomics remains limited due to the availability of AF. Even in the two studies with AF listed in Table 1, the number of CHD cases was small (16, 17). AF contains amniotic epithelial cells and numerous metabolites produced by fetal and placenta tissues, which can reflect pathological condition during fetal development (21). As a basic study, AF in the second trimester was selected for analysis, and the main component of AF was fetal urine in the second trimester (22). Compared with blood and urine of pregnant woman, AF was less affected by factors and its composition was relatively simple, so it was beneficial for us to search for markers. Although AF acquisition is invasive and not suitable for widespread use, if we can confirm a metabolic profiling difference of CHD in AF and find some effective markers, this will guide us to further explore CHD markers in blood of pregnant woman, and to explore a detective method, which is even suitable for a wider population. Untargeted metabolomics provides a comprehensive analysis of all endogenous metabolites, therefore, it is feasible to utilize AF and metabolomics methods to detect CHD. In this study, global metabolite profiles were analyzed in 71 AF samples from CHD pregnancies. Alterations in AF metabolites and biomarkers of CHD were analyzed, which may provide important clue in understanding the pathogenesis of CHD and developing detective markers of CHD.

**Table 1:** CHD-related metabolomics studies have been reported during pregnancy.

**Year**	**Reference**	**Sample type**	**CHD number**	**Detection method**	**Differential metabolites**	**Proposed dignostic biomarkers**
2009	Graça et al. (16)	Amniotic fluid	12(fetal malformations including CHD)	NMR and Enzymatic assays	20(organic acid, amino acid, carbohydrate, protein, ammonia, urea)	NO
2010	Graça et al. (17)	Amniotic fluid	27(fetal malformations including CHD)	NMR	22(organic acid, amino acid, carbohydrate, protein, and so on)	NO
2014	Bahado-Singh et al. (18)	Maternal serum	27	DI/LC-MS/MS and NMR	123(acylcarnitine, sphingomyelin, acetate, acetone, ethanol, pyruvate, 3-methylhistidine)	C3-OH, C5:1-DC, C14:2-OH
2019	Xie et al. (19)	Maternal urinary	70	GC-MS	20(organic acid, saccharides, amino acid, uracil)	4-hydroxybenzeneacetic acid, 5-trimethylsilyloxy-n-valeric acid, propanedioic acid, hydracrylic acid, uric acid
2020	Friedman et al. (20)	Maternal urinary	36	Tandem mass spectrometry and NMR	23(organic acid, amino acid, carbohydrate, Choline, carnitine, and so on)	histamine, choline, glucose, formate, methionine, carnitine, ultrasound 4-chamber view

*CHD, congenital heart disease; NM*.

*R, nuclear magnetic resonance; DI/LC-MS/MS, direct injection/liquid chromatography and tandem mass spectrometry; GC-MS, gas chromatography-mass spectrometry; C3-OH, hydroxypropionylcarnitine; C5:1-DC, glutaconylcarnitine; C14:2-OH, hydroxytetradecadienylcarnitine*.

## Materials and Methods

### Samples

Ethical approval of this study was obtained from the Medical Ethics Committee of Nanjing Maternity and Child Health Care Hospital and a waiver of informed consent was approved. This study was a retrospective analysis. AF samples used in this study were collected from residual AF of patients visiting the prenatal diagnosis center at the Nanjing Maternity and Child Health Care Hospital between January 2012 and December 2018; the specimens were stored at −80°C. Among the 1,859 AF samples, 71 cases of CHD and 149 control samples were selected. The cases of CHD were single pregnancies from the Jiangsu province diagnosed as CHD by fetal echocardiogram and referred to our prenatal diagnosis center. In 30 cases of CHD, the pregnancy was terminated. The parents did not consent to autopsy due to cultural norms; therefore, CHD diagnosis was based on prenatal echocardiography only. In the 41 cases of CHD that resulted in a live birth, postnatal ultrasound diagnosis was consistent with prenatal results. Cases with structural malformations other than CHD, chromosomal abnormalities, and pregnancy complications such as hypertension and diabetes were excluded. Control samples corresponded to normal single pregnancies with normal fetuses in which amniocentesis was performed due to advanced age or an abnormal result of serological screening for Down syndrome in the second trimester. The patients were from the same geographic regions as the CHD cases, and postpartum follow-up of these pregnancies was normal. Cases of CHD or other structural malformations, chromosomal abnormalities, pregnancy complications, such as hypertension and diabetes, were excluded from the control group.

The selected samples were divided into two independent sets: the discovery set and the validation set. The discovery set of AF samples consisted of 18 CHD samples and 35 controls sample, while the validation set consisted of 53 CHD samples and 114 controls. CHD was classified according to the method of Botto et al. (23) (Table 2).

**Table 2:** Classification of CHD in the discovery set and the validation set.

**CHD classification**		**Number of cases in the discovery set**	**Number of cases in the validation set**
**Conotruncal**			
	IAA, B	0	7
	d-TGA	0	3
	TOF	1	6
	DORV	0	3
	Truncus arteriosus	0	2
	PA-VSD (TOF anatomy)	0	1
**Heterotaxy**		1	4
**LVOTO**	
	HLHS	0	1
	HLHS+VSD	0	1
	HLHS+PAVR	0	1
**Septal Defects**	VSD	15	15
**Septal** **+** **RVOTO**	
	PVS+ASD+VSD	0	1
	VSD +PVS	0	2
**Single ventricle/complex**	
	Multiple, complex heart anomaly	1	3
**Other association**		0	3

*IAA, B, interrupted aortic arch type B; d-TGA, d-transposition of the great arteries; TOF, tetralogy of fallot; DORV, double outlet right ventricle; PA, pulmonary atresia; VSD, ventricular septal defect; LVOTO, left ventricular outflow tract obstruction; HLHS, hypoplastic left heart syndrome; PAVR, partial anomalous pulmonary venous return; RVOTO, right ventricular outflow tract obstruction; ASD, atrial septal defect; PVS, pulmonary valve stenosis*.

In order to detect CHD serum biomarkers, 29 CHD and 58 control cases with matched GA, maternal age and BMI (body mass index) were selected from 86,120 samples we saved after serological screening for Down syndrome in the second trimester in our center from August 2016 to December 2019 (Table 3). Fetal information and all outcomes were obtained through follow-up. Other selection and exclusion criteria were basically the same as those we used for the selection of AF samples above.

**Table 3:** Demographic characteristics of samples used for maternal serum uric acid analysis.

**Subject characteristics**	**Control**	**CHD**	***P* value**
Number of samples	58	29	
Maternal age (years)	28 (23–34)	28 (22–34)	0.762
Gestational age (weeks)	17 (15–18)	17 (15–18)	0.526
Body mass index (kg/m^2^)	21.99 (17.1–33.59)	22.15 (17.30–32.39)	0.829

### Reagents and Instruments

Methoxyamine HCl, fatty acid methyl ester (C7–C30, FAMEs) standards, pyridine, and anhydrous sodium sulfate were obtained from Sigma-Aldrich (St. Louis, MO, USA). N-methyl-N(trimethylsilyl)trifluoroacetamide (MSTFA) with 1% (vol/vol) trimethylchlorosilane (TMCS), methanol (Optima LC-MS), acetonitrile (Optima LC-MS), dichloromethane, hexane, chloroform, and acetone were purchased from Thermo-Fisher Scientific (FairLawn, NJ, USA). Ultrapure water was produced by a Milli-Q reference system equipped with a LC-MS Pak filter (Millipore, Billerica, MA, USA). A time-of-flight mass spectrometry (GC-TOF/MS) system (Pegasus HT, Leco Corp., St. Joseph, MO, USA) was equipped with an Agilent 7890B gas chromatography and a Gerstel multipurpose sample MPS2 with dual heads (Gerstel, Muehlheim, Germany). Separation was achieved on a Rxi-5 ms capillary column (30 m × 250 μm i.d., 0.25 μm film thickness, Restek Corporation, Bellefonte, PA, USA). A Beckman Coulter AU5800 automatic biochemical analyzer (Brea, CA, USA) and a uric acid biochemical assay kit [DiaSys Diagnostic Systems (Shanghai) Co., Ltd., Shanghai, China] were used for serum uric acid detection.

### Sample Preparation for GC/TOF-MS

The metabolomics analysis was performed on XploreMET platform (Metabo-Profile, Shanghai, China) as previously described (24, 25). Briefly, the frozen AF samples were thawed on ice and centrifuged at 1,000 g for 3 min at 4 °C. Each aliquot of 100 μL of AF sample was pipetted into a precooled EP tube, and 10 μL of internal standard solution was added. Each 200 μL of precooled methanol:chloroform (3:1, v/v) was used for metabolite extraction. After centrifugation at 13,000 g for 20 min at 4°C, 200 μL of supernatant was carefully transferred into an autosampler vial (Agilent Technologies, Foster City, CA, USA). The remaining supernatant from each sample was pooled to make quality control (QC) samples. All the samples in autosampler vials were centrifuged for 5 min in a vacuum centrifuge concentrator (Labconco, Kansas City, MO, USA) to remove the chloroform solvent, transferred to a freeze dryer (Labconco, Kansas City, MO, USA) and completely lyophilized. Dichloromethane was added to the lyophilized residue to ensure complete dryness of the sample. The dried powder was put at room temperature and filled with high-purity nitrogen (Parker Balston, Lancaster, NY, USA). The sample derivatization and injection were performed by a robotic multipurpose sample MPS2 with dual heads (Gerstel, Muehlheim, Germany) as follows: the addition of 50 μL of methoxyamine (20 mg/mL in pyridine) to the dried sample at 30°C for 2 h, followed by the addition of 50 μL of MSTFA (1% TMCS) containing FAMEs, and mixing at 37.5°C for 1 h. In parallel, the derivatized samples were injected with sample injection head after derivatization.

### GC/TOF-MS Analysis Conditions

The 5% bonded and cross-linked diphenyl/95% dimethyl polysiloxane (Agilent J&W Scientific, Folsom, CA, USA) with helium (99.9999%) was used as the carrier gas at a constant flow rate of 1.0 mL/min. The temperature of both the injection and transfer interfaces was 270°C. The injection volume was 1 μL. The GC temperature programming was set to 2 min isothermal heating at 80°C, followed by 12°C/min oven temperature ramps to 80–300°C, 4.5 min to 300°C, and 40°C/min to 300–320°C, and final maintenance at 320°C for 1 min. In the TOF MS setting, the measurements were made using electron impact ionization (70 eV) in the full scan mode (m/z 50–500), ion source was set to 220°C, with an acquisition rate of 25 spectra/s, and the mass range was set to 50–500 Da. QC sample was analyzed across the entire sample set for quality control of the data.

### Quantitative Analysis of Serum Uric Acid Level

The frozen serum samples were dissolved in a refrigerator at 4°C. After fully mixing, 500 μL serum was pipetted into a cuvette. Place the cuvette on a cuvette holder, and then uric acid was detected by a Beckman Coulter AU5800 automatic biochemical analyzer and a uric acid biochemical assay kit according to the manufacturer's protocol. Standards, reagent blank and quality control samples were analyzed at the same time, then uric acid concentration of the samples was calculated according to the standard curve. The basic principle of uric acid analysis was as follows: (1) uric acid reacted with uricase to produce hydrogen peroxide, carbon dioxide and allantoin; (2) through the action of oxidase, hydrogen peroxide reacted with 4-aminoantipyrine and N-ethyl-N-(hydroxy-3-sulfopropyl)-m-toluidine (TOOS) to produce blue-purple indamide; (3) the absorbance value was measured at 550 nm.

### Data Analysis

Raw data were analyzed by XploreMET 3.0 software, including automated baseline denosing, peak picking, deconvolution and signal alignment (24). Metabolite annotation was performed by comparing the retention indices and mass spectral data with JiaLib metabolite database (26, 27). Each data set was transformed into the comparable data vectors and the orthogonal partial least square discrimination analysis (OPLS-DA) model was performed by MetaboAnalyst 5.0. *Mann-Whitney U test, Student's t-test*, logistic regression analysis and receiver operating characteristic (ROC) curve were performed by R language, and *P* < 0.05 was considered significant. GraphPad Prism version 6 was used for drawing box diagram.

## Results

### Study Population

Maternal age of CHD and control cases in the discovery set ranged from 23 to 37 years (median, 29.5) and from 20 to 37 years (median, 29), respectively. Corresponding values in the validation set were 22 to 39 years (median, 28) and 21 to 42 years (median, 31) years. Gestational age (GA) of CHD and control cases in the discovery set ranged from 23 to 30 weeks (median, 27) and 18 to 28 weeks (median, 20), respectively. Corresponding values in the validation set were 20 to 26 weeks (median, 24) and 17 to 24 weeks (median, 20). The numbers of female/male CHD and control fetuses were, respectively, 10/8 and 16/19 in the discovery set and 20/33 and 65/49 in the validation set. There were no statistically significant differences in the maternal age (*P* = 0.972) and fetal gender (*P* = 0.497) between CHD and control cases in the discovery set, but GA was significantly different (*P* < 0.001). There were statistically significant differences in the maternal age (*P* < 0.001), fetal gender (*P* = 0.020), and GA (*P* < 0.001) between CHD and control cases in the validation set. Except for terminated pregnancies, all CHD cases were diagnosed postpartum, and all control samples were CHD-free.

### Metabolic Profiles of AF

**Figure 1:**
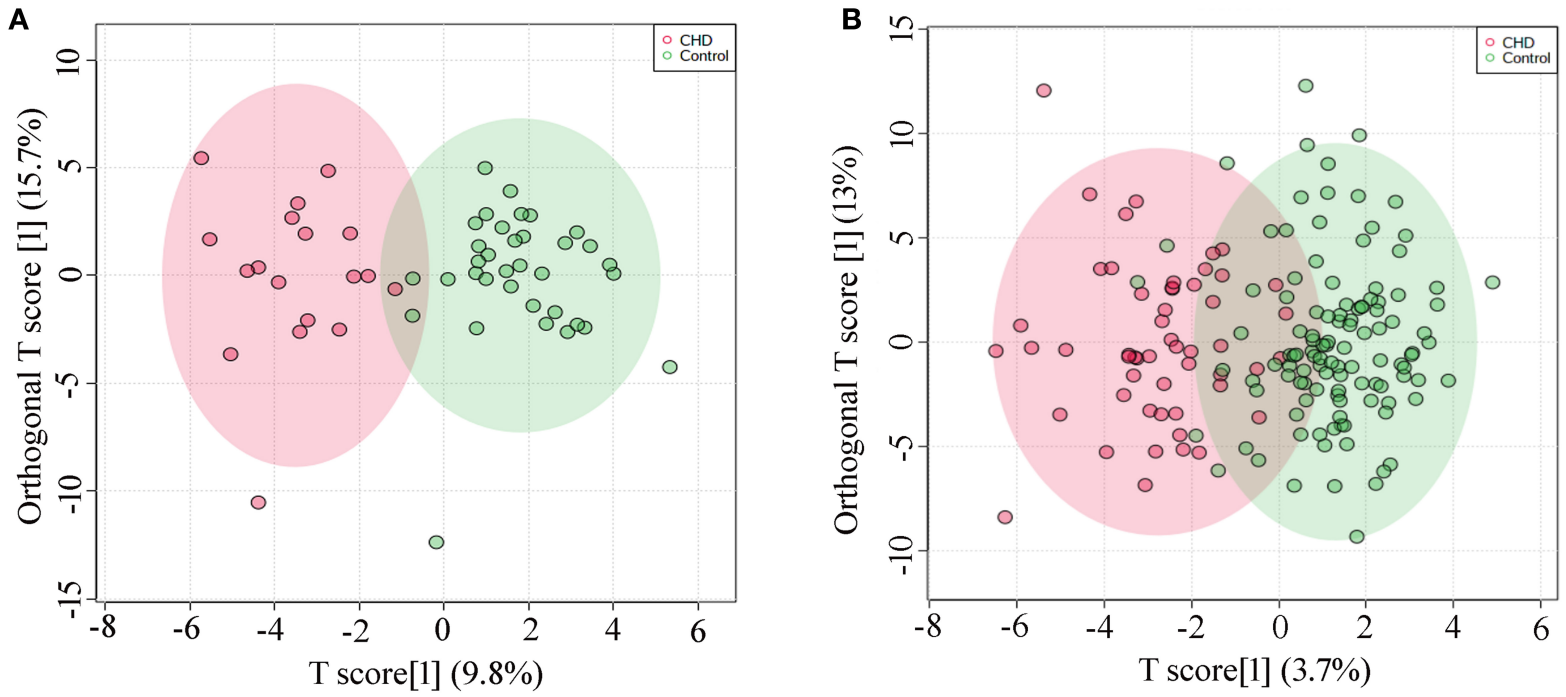
OPLS-DA analysis **(A)** Score plot of the discovery set, R^2^Y = 0.787, Q^2^Y = 0.520; **(B)** Score plot of the validation set, R^2^Y = 0.651, Q^2^Y = 0.469.

Metabolic profiles of AF samples from CHD and control of the discovery set and the validation set were analyzed by OPLS-DA model. A clear separation of the metabolic profiles between CHD and the control group could be observed in the discovery set (R^2^Y = 0.787, Q^2^Y = 0.520, Figure 1A), and in the validation set (R^2^Y = 0.651, Q^2^Y = 0.469, Figure 1B). In addition, permutation testing was used to validate the classification model, and *P*-values of the discovery set and the validation set were both significant (*P* < 5e^−04^).

### Biomarker Selection of CHD

To identify potential biomarkers, we adjusted the metabolic profiles for gestational age, maternal age, and fetal gender using logistic regression analysis. This approach identified a total of 9 differential metabolites in the discovery set (Supplementary Table 1). Subsequently, we verified 2 differential metabolites, i.e., uric acid and proline, in the validation set (Supplementary Table 2), both showing increased levels in maternal AF of CHD cases (Table 4). The odds ratio (OR) values of uric acid and proline were, respectively, 7.69 (95% CI: 1.18–50.13) and 4.42 (95% CI: 1.12–17.45) in the discovery set, and 3.24 (95% CI: 1.62–6.48) and 2.20 (95% CI: 1.20–4.03) in the validation set. These results showed that uric acid and proline were positively correlated with the risk of CHD.

**Table 4:** Verified CHD differential metabolites.

**Class**	**Metabolite**	**HMDB ID**	**Trend in CHD**	**Discovery set**		**Validation set**	
				**OR(95% CI)**	***P*-value^a^**	**OR(95% CI)**	***P*-value^a^**
Amino Acid	Proline	HMDB0000162	↑	4.42(1.12–17.45)	0.034	2.20(1.20–4.03)	0.011
Organic Acid	Uric acid	HMDB0000289	↑	7.69(1.18–50.13)	0.033	3.24(1.62–6.48)	0.001

*HMDB, human metabolome database; OR, odd ratio; CI, confidence interval*.

a *Logistic regression analysis adjusted for gestational age, maternal age, and fetal gender*.

**Figure 2:**
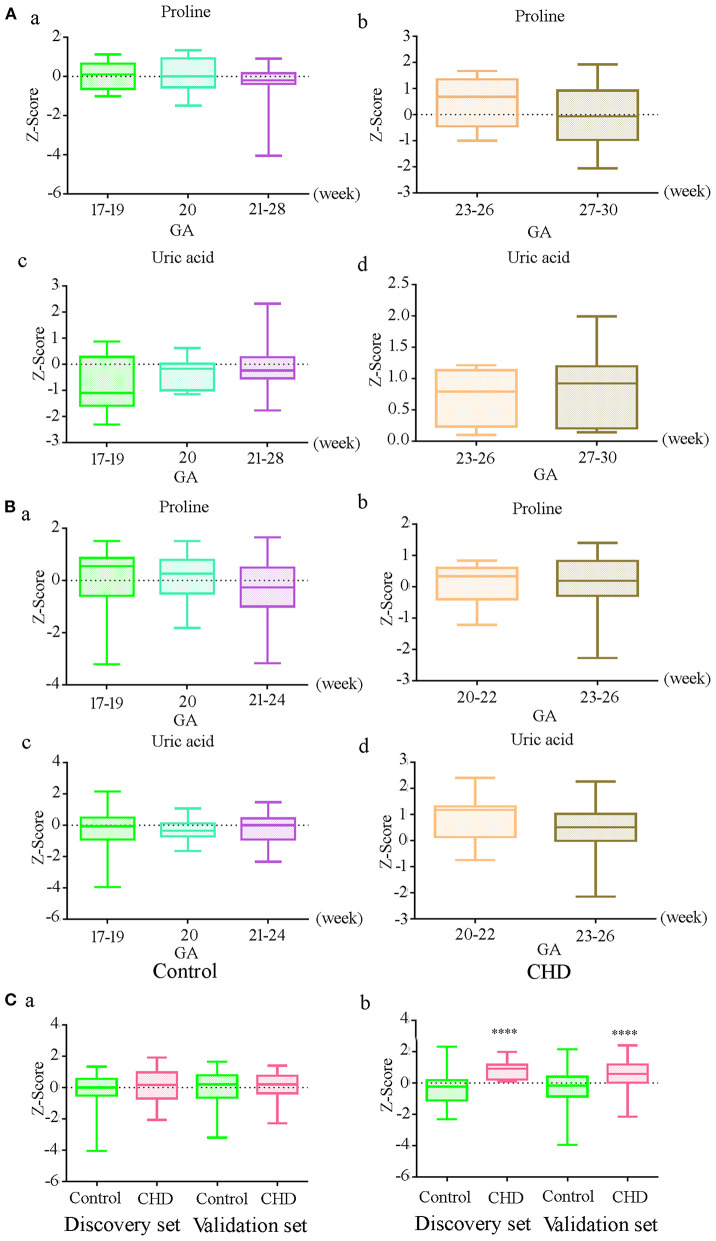
Relationship between gestational age (GA) and differential metabolite levels before correction of GA, maternal age and fetal gender **(A)** Levels of proline and uric acid at different gestational in controls (a, c) and CHDs (b, d) in the discovery set; **(B)** Levels of proline and uric acid at different gestational in controls (a, c) and CHDs (b, d) in the validation set; **(C)** Proline (a) and uric acid (b) levels in controls and CHDs regardless of GA. **** means *P* < 0.0001. The Y axis shows the Z-Score standardized value of peak intensity, Z-Score = (original data—mean)/standard deviation.

To further explore the effect of GA on uric acid and proline levels, we analyzed the original data without the adjustment for GA, maternal age, and fetal gender. The results demonstrated that there were no significant differences in the level of uric acid and proline at different gestational weeks in controls in the discovery and the validation sets (Figures 2Aa,c, Ba,c) (*P* > 0.05). The absence of differences was also observed in CHD cases (Figures 2Ab,d, Bb,d). There was no significant difference in the proline level between controls and CHD (Figure 2Ca). However, the uric acid level was significantly elevated in CHD cases (Figure 2Cb) in both sets. These results indicated that there was no significant relationship between proline and uric acid levels and GA within the range we investigated.

### Receiver Operator Characteristic Curve Analysis

**Figure 3:**
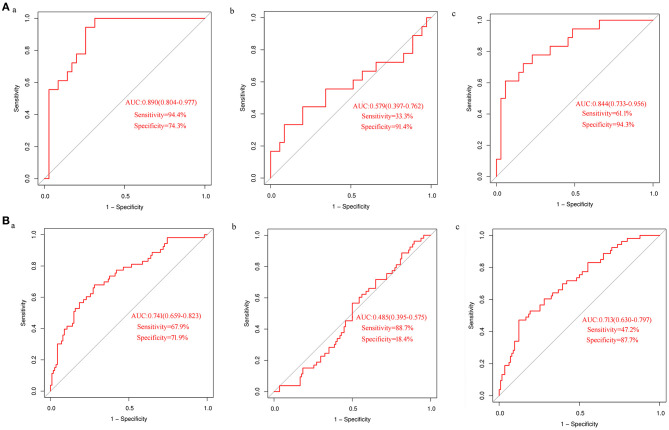
ROC curves analysis of differential metabolites in detecting CHD **(A)** ROC curve analysis of uric acid (a) and proline alone (b) or in combination (c) to detect CHD in the discovery set; **(B)** ROC curve analysis of uric acid (a) and proline alone (b) or in combination (c) to detect CHD in the validation set.

In order to test the performance of the verified differential metabolites in predicting CHD, we performed ROC curve analysis for each indicator and their combination in detecting CHD. ROC curves of uric acid in the discovery set (Figure 3Aa) and the validation set (Figure 3Ba) showed a moderate predictive power; the area under curve (AUC) was 0.890 (95% CI: 0.804–0.977) and 0.741 (95% CI: 0.659–0.823), sensitivity was 94.4 and 67.9%, and specificity was 74.3 and 71.9%, respectively. The ability of proline to detect CHD was low; the AUC of the discovery set and the validation set was 0.579 (95% CI: 0.397–0.762) and 0.485 (95% CI: 0.395–0.575), the sensitivity was 33.3 and 88.7%, and the specificity was 91.4 and 18.4%, respectively (Figures 3Ab, Bb). Logistic regression analysis demonstrated that the combination of proline and uric acid is not as good predictor of CHD as uric acid alone. The AUCs of the discovery set (Figure 3Ac) and the validation set (Figure 3Bc) were 0.844 (95% CI: 0.733–0.956) and 0.713 (95% CI: 0.630–0.797), respectively. These results indicated that uric acid might be a potential biomarker of CHD in AF.

### Analysis of Uric Acid Levels in Maternal Serum of CHD Cases

**Figure 4:**
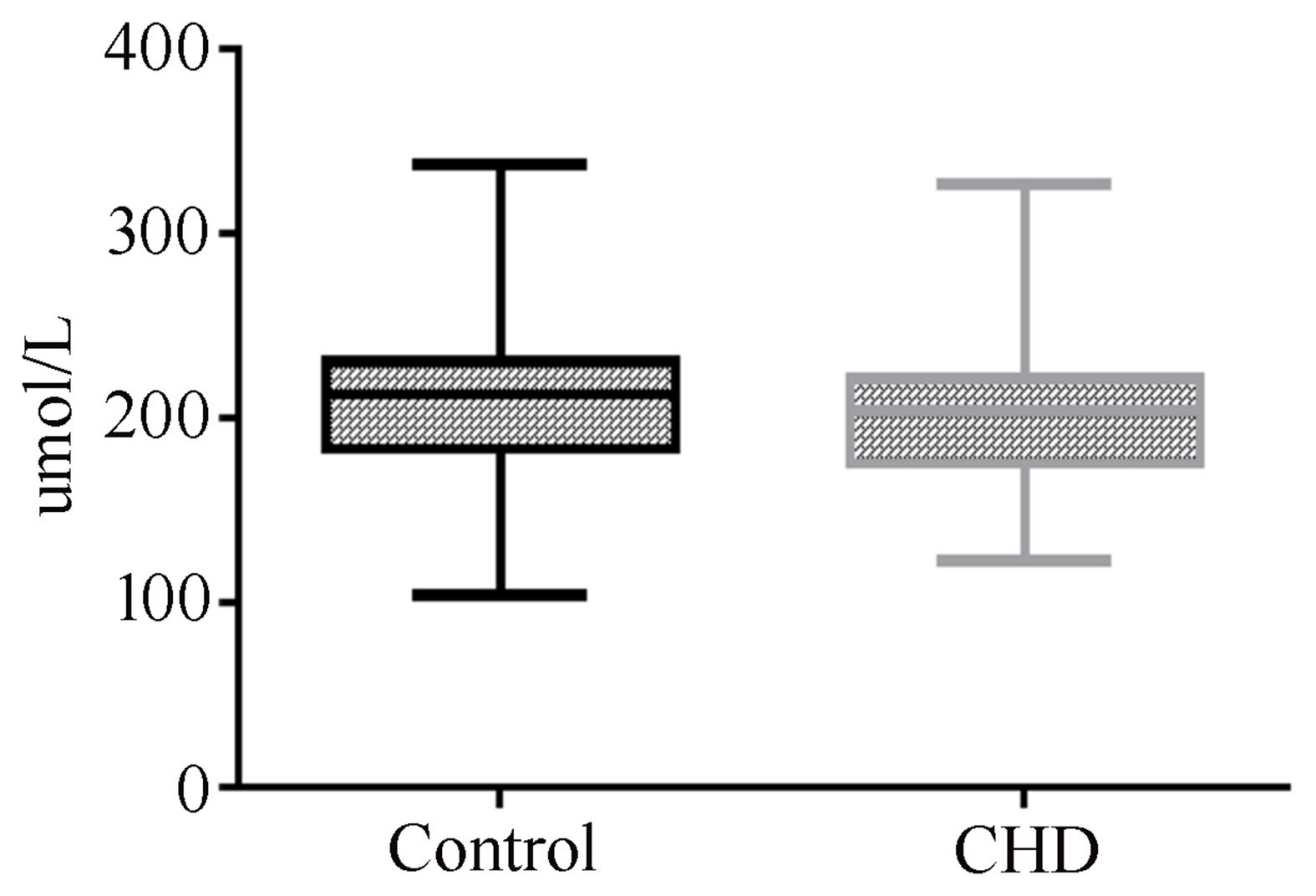
Maternal serum uric acid concentration in normal or CHD pregnancies during the second trimester.

Uric acid can be used as a biomarker for the detection of CHD in AF. However, due to the invasive mode of AF collection, it is not suitable as a marker in the general population. Therefore, we measured the levels of uric acid in maternal serum in CHD cases during the second trimester. Twenty-nine maternal serum samples of CHD cases and 58 control samples matched for GA, maternal age, and maternal BMI were selected (Table 3). However, we did not detect a significant difference in uric acid levels between control and CHD samples (Figure 4).

## Discussion

The changes in specific metabolites in AF are closely related to fetal malformations (28). In this study, we selected samples in the second trimester of pregnancy for metabolomics study, because, at this time, fetal organogenesis period almost ended and the composition was relatively simple, mainly fetal urine, making it easier for us to seek for differential metabolites, while untargeted metabolomics method made it possible. Thus, in this study we selected AF samples to analyze metabolites related to fetal CHD by GC-TOF-MS based metabolomics method. Published studies used NMR to explore AF metabolism (16, 17).

The OPLS-DA model showed that there was a significant difference in metabolic profiles between CHD and control cases in AF, with obvious separation trend. In univariate analysis, only changes in the level of uric acid and proline were verified in both sets, and the concentration of both metabolites was significantly elevated in CHD. Of relevance, there was no significant difference in proline level between controls and CHD before the adjustment for GA, maternal age, and fetal gender, and the difference appeared only after adjusting for these factors. Conversely, the higher level of uric acid CHD was apparent before and after the adjustment. In addition, we believe that maternal age and fetal gender have no significant effect on proline and uric acid levels. This conviction is based on the fact that regardless of the difference between the two groups, the changes in proline and uric acid levels were consistent across the discovery and validation sets.

Graca et al. (16, 17) did not find changes in proline and uric acid levels in AF when using MRI to detect fetal malformations, including CHD. However, the changes in uric acid were consistent with the urine biomarker reported by Xie et al. (19), as shown in Table 1. Our results using AF were not consistent with those obtained using the maternal serum in early pregnancy reported by Bahado-Singh et al. (18) and our own results in maternal serum. These discrepancies may be caused by the selected sample type because our study of the serum of pregnant women in the second trimester also did not detect a significant change in uric acid in the CHD cases. Although uric acid in AF can be used as a marker for CHD, uric acid in maternal serum is not a suitable marker for this condition. A previous study by Ke et al. (29) showed that CHD pregnancies had a higher probability of hyperuricemia than non-CHD pregnancies, and uric acid played an important role in the early development of the heart. They concluded that uric acid might be a novel marker for CHD. However, our results documented that serum levels of uric acid in pregnant women with CHDs were all within the normal range (< 430 μmol/L), while in the study of Ke et al. (29), among 306 CHD pregnancies, only 43 (14.05%) had hyperuricemia. Therefore, we considered that uric acid in the blood is not a biomarker for CHD.

To evaluate the performance of proline and uric acid in detecting CHD, we performed the ROC curve analysis. The results showed that uric acid had moderate predictive power, while the AUC value for the ability of proline to detect CHD was low (AUC < 0.6). Based on AUC, the performance of the combination of uric acid and proline in detecting CHD did not exceed that of uric acid alone; therefore, the level of uric acid in AF may be a potential marker for CHD. Uric acid was closely related to CHD, and the OR values in the discovery set and the validation set were 7.69 (95% CI: 1.18–50.13) and 3.24 (95% CI: 1.62–6.48), respectively. While the collection of AF is invasive and not suitable for widespread use, the uric acid test in the AF can be employed as an additional test after amniocentesis, assisting in prenatal testing for CHD.

Uric acid may play an important role in the pathological process of CHD. Uric acid is the end product of purine metabolism in humans (30). Ke et al. (29) reported that uric acid can enhance cardiac differentiation of human pluripotent stem cells (PSCs) at day 0–2 through SNAI pathway-mediated epithelial–mesenchymal transition (EMT) and a lengthened G0/G1 phase, indicating the importance of uric acid in heart development. Uric acid is considered a risk factor for cardiovascular disease, and elevated serum uric acid is associated with increased risk of incident heart failure (30, 31). Our results also showed that the level of uric acid in CHD was significantly increased in AF. It can be seen that, on the one hand, uric acid promotes the development of the heart, and on the other hand, high levels of uric acid are harmful to the heart. It is possible that the effect of uric acid on the heart depends on its level, and when it exceeds a certain level, it may have an adverse effect.

Our study first identified uric acid as an effective biomarker for CHD in AF and this will provide more opportunity to explore the pathogenesis of CHD and to develop detective biomarkers of CHD. However, Our study has some limitations. Firstly, from the perspective of sample selection, we chose AF sample, which is invasive and not suitable for routine clinical use. However, the composition of AF is relatively simple compared with blood and urine, and it is easy to explore the markers of CHD. As a basic study, it lays the foundation for our future study on blood metabolomics of pregnant women. What's more, heterogeneity may be caused by various types of CHD. This requires us to explore the differences between different CHDs in the next step. Given that the concentration of uric acid is increased in CHD, we believe that uric acid may be involved in the pathogenesis of CHD. We will explore this possibility and its underlying mechanism in future studies.

## Data Availability Statement

The original contributions presented in the study are included in the article/Supplementary Material, further inquiries can be directed to the corresponding author/s.

## Ethics Statement

The studies involving human participants were reviewed and approved by Medical Ethics Committee of Nanjing Maternity and Child Health Care Hospital. Written informed consent for participation was not required for this study in accordance with the national legislation and the institutional requirements.

## Author Contributions

YL, YS, LY, MH, and XZ were responsible for comprehensive sample collection, data collection, experimental operation, data analysis, and paper writing. XW, XG, PY, YW, and LM were responsible for sample detection. RZ, XZ, and CL were responsible for amniotic fluid sample collection and information collection. ZX, TJ, and PH were responsible for the funding, research design, and paper writing. All authors contributed to the article and approved the submitted version.

## Conflict of Interest

The authors declare that the research was conducted in the absence of any commercial or financial relationships that could be construed as a potential conflict of interest.

## References

[1] Giang KW Mandalenakis Z Dellborg M Lappas G Eriksson P Hansson PO. Long-term risk of hemorrhagic stroke in young patients with congenital heart disease. Stroke. (2018) 49:1155–62. 10.1161/STROKEAHA.117.020032 29626133 PMC5916472

[2] Zhao QM Liu F Wu L Ma XJ Niu C Huang GY. Prevalence of congenital heart disease at live birth in china. J Pediatr. (2019) 204:53–8. 10.1016/j.jpeds.2018.08.040 30270157

[3] Pérez-Lescure Picarzo J Mosquera González M Latasa Zamalloa P Crespo Marcos D. Congenital heart disease mortality in Spain during a 10 year period (2003-2012). An Pediatr (Barc). (2018) 88:273–9. 10.1016/j.anpede.2017.06.003 28711428

[4] Yang Q Wu F Mi Y Wang F Cai K Yang X. Aberrant expression of miR-29b-3p influences heart development and cardiomyocyte proliferation by targeting NOTCH2. Cell Prolif. (2020) 53:e12764. 10.1111/cpr.12764 32077168 PMC7106969

[5] Leung KKY Hon KL Yeung A Leung AKC Man E. Congenital infections in Hong Kong: an overview of TORCH. Hong Kong Med J. (2020) 26:127–38. 10.12809/hkmj198287 32245914

[6] Wong P Denburg A Dave M Levin L Morinis JO Suleman S. Early life environment and social determinants of cardiac health in children with congenital heart disease. Paediatr Child Health. (2018) 23:92–5. 10.1093/pch/pxx146 29686491 PMC5905484

[7] Zhang H Liu L Tian J. Molecular mechanisms of congenital heart disease in down syndrome. Genes Dis. (2019) 6:372–7. 10.1016/j.gendis.2019.06.007 31832516 PMC6889238

[8] Wu MH Chen HC Lu CW Wang JK Huang SC Huang SK. Prevalence of congenital heart disease at live birth in Taiwan. J Pediatr. (2010) 156:782–5. 10.1016/j.jpeds.2009.11.062 20138303

[9] Lisanti AJ Vittner D Medoff-Cooper B Fogel J Wernovsky G Butler S. Individualized family-centered developmental care: an essential model to address the unique needs of infants with congenital heart disease. J Cardiovasc Nurs. (2019) 34:85–93. 10.1097/JCN.0000000000000546 30303895 PMC6283700

[10] Kaugars A Shields C Brosig C. Stress and quality of life among parents of children with congenital heart disease referred for psychological services. Congenit Heart Dis. (2018) 13:72–8. 10.1111/chd.12547 29071790

[11] Chew C Halliday JL Riley MM Penny DJ. Population-based study of antenatal detection of congenital heart disease by ultrasound examination. Ultrasound Obstet Gynecol. (2007) 29:619–24. 10.1002/uog.4023 17523161

[12] Pinto NM Keenan HT Minich LL Puchalski MD Heywood M Botto LD. Barriers to prenatal detection of congenital heart disease: a population-based study. Ultrasound Obstet Gynecol. (2012) 40:418–25. 10.1002/uog.10116 21998002

[13] Nicholson JK Lindon JC Holmes E. 'Metabonomics': understanding the metabolic responses of living systems to pathophysiological stimuli via multivariate statistical analysis of biological NMR spectroscopic data. Xenobiotica. (1999) 29:1181–9. 10.1080/004982599238047 10598751

[14] Beale DJ Pinu FR Kouremenos KA Poojary MM Narayana VK Boughton BA. Review of recent developments in GC-MS approaches to metabolomics-based research. Metabolomics. (2018) 14:152. 10.1007/s11306-018-1449-2 30830421

[15] Percival BC Gibson M Wilson PB Platt FM Grootveld M. Metabolomic studies of lipid storage disorders, with special reference to Niemann-Pick Type C disease: a critical review with future perspectives. Int J Mol Sci. (2020) 21:2533. 10.3390/ijms21072533 32260582 PMC7178094

[16] Graça G Duarte IF Barros AS Goodfellow BJ Diaz S Carreira IM. (1)H NMR based metabonomics of human amniotic fluid for the metabolic characterization of fetus malformations. J Proteome Res. (2009) 8:4144–50. 10.1021/pr900386f 19453159

[17] Graca G Duarte IF Barros AS Goodfellow BJ Diaz SO Pinto J. Impact of prenatal disorders on the metabolic profile of second trimester amniotic fluid: a nuclear magnetic resonance metabonomic study. J Proteome Res. (2010) 9:6016–24. 10.1021/pr100815q 20849080

[18] Bahado-Singh RO Ertl R Mandal R Bjorndahl TC Syngelaki A Han B. Metabolomic prediction of fetal congenital heart defect in the first trimester. Am J Obstet Gynecol. (2014) 211:240.e1–14. 10.1016/j.ajog.2014.03.056 24704061

[19] Xie D Luo Y Xiong X Lou M Liu Z Wang A. Study on the potential biomarkers of maternal urine metabolomics for fetus with congenital heart diseases based on modified gas chromatograph-mass spectrometer. Biomed Res Int. (2019) 2019:1905416. 10.1155/2019/1905416 31198782 PMC6526572

[20] Friedman P Yilmaz A Ugur Z Jafar F Whitten A Turkoglu O. Maternal urine metabolomics and the prediction of fetal non-syndromic congenital heart defect (CHD). Am J Obstet Gynecol. (2020) 222:S79. 10.1016/j.ajog.2019.11.113

[21] Shan J Xie T Xu J Zhou H Zhao X. Metabolomics of the amniotic fluid: is it a feasible approach to evaluate the safety of Chinese medicine during pregnancy? J Appl Toxicol. (2019) 39:163–71. 10.1002/jat.3653 29931825

[22] Gomez-Lopez N Romero R Xu Y Miller D Leng Y Panaitescu B. The immunophenotype of amniotic fluid leukocytes in normal and complicated pregnancies. Am J Reprod Immunol. (2018) 79:e12827. 10.1111/aji.12827 29500850 PMC5951617

[23] Botto LD Lin AE Riehle-Colarusso T Malik S Correa A. Seeking causes: classifying and evaluating congenital heart defects in etiologic studies. Birth Defects Res A Clin Mol Teratol. (2007) 79:714–27. 10.1002/bdra.20403 17729292

[24] Ni Y Qiu Y Jiang W Suttlemyre K Su M Zhang W. ADAP-GC 2.0: deconvolution of coeluting metabolites from GC/TOF-MS data for metabolomics studies. Anal Chem. (2012) 84:6619–29. 10.1021/ac300898h 22747237

[25] Chen WL Wang YY Zhao A Xia L Xie G Su M. Enhanced fructose utilization mediated by SLC2A5 is a unique metabolic feature of acute myeloid leukemia with therapeutic potential. Cancer Cell. (2016) 30:779–91. 10.1016/j.ccell.2016.09.006 27746145 PMC5496656

[26] Jiang W Qiu Y Ni Y Su M Jia W Du X. An automated data analysis pipeline for GC-TOF-MS metabonomics studies. J Proteome Res. (2010) 9:5974–81. 10.1021/pr1007703 20825247

[27] Wang Y Zhao H Liu Y Guo W Bao Y Zhang M. GC-MS-based metabolomics to reveal the protective effect of gross saponins of *Tribulus terrestris* fruit against ischemic stroke in rat. Molecules. (2019) 24:793. 10.3390/molecules24040793 30813246 PMC6412276

[28] Palmas F Fattuoni C Noto A Barberini L Dessì A Fanos V. The choice of amniotic fluid in metabolomics for the monitoring of fetus health. Expert Rev Mol Diagn. (2016) 16:473–86. 10.1586/14737159.2016.1139456 26760526

[29] Ke B Zeng Y Zhao Z Han F Liu T Wang J. Uric acid: a potent molecular contributor to pluripotent stem cell cardiac differentiation via mesoderm specification. Cell Death Differ. (2019) 26:826–42. 10.1038/s41418-018-0157-9 30038385 PMC6461775

[30] Ndrepepa G. Uric acid and cardiovascular disease. Clin Chim Acta. (2018) 484:150–63. 10.1016/j.cca.2018.05.046 29803897

[31] Huang G Qin J Deng X Luo G Yu D Zhang M. Prognostic value of serum uric acid in patients with acute heart failure: a meta-analysis. Medicine (Baltimore). (2019) 98:e14525. 10.1097/MD.0000000000014525 30813158 PMC6408052

