# Supplementary material for: Analysis of Biomarkers for Congenital Heart Disease Based on Maternal Amniotic Fluid Metabolomics
Source: Front Cardiovasc Med. 2021 Jun 7;8:671191. doi: 10.3389/fcvm.2021.671191 (PMC8215886; doi:10.3389/fcvm.2021.671191)
Supplement: Supplementary file 1 [file Table_1.DOCX]

Table S1. Differential metabolites of CHD in the discovery set identified after adjustment for gestational week, maternal age, and fetal gender.

| Class | Metabolite | HMDB ID | Trend in CHD | OR(95% CI) | *P*-value^a^ |
| --- | --- | --- | --- | --- | --- |
| Amino acids | Proline | HMDB0000162 | ↑ | 4.42(1.12-17.45) | 0.034 |
|  | Glutiamne | HMDB0000641 | ↑ | 11.89(1.15-123.31) | 0.038 |
|  | Alloisoleucine | HMDB0000557 | ↓ | 4.47(1.02-19.65) | 0.047 |
|  | Alanine | HMDB0000161 | ↓ | 6.94(1.00-47.96) | 0.049 |
|  | Serine | HMDB0000187 | ↑ | 9.77(1.00-95.12) | 0.049 |
| Organic Acids | Uric acid | HMDB0000289 | ↑ | 7.69(1.18-50.13) | 0.033 |
|  | Malonic acid | HMDB0000691 | ↑ | 0.26(0.07-0.95) | 0.042 |
|  | p-Hydroxyphenylacetic acid | HMDB0000020 | ↑ | 3.99(1.03-15.49) | 0.045 |
|  | Oxalic acid | HMDB0002329 | ↓ | 0.34(0.11-1.00) | 0.049 |

HMDB,Human Metabolome Database; OR, odd ratio;

^a^ Logistic regression analysis adjusted for gestational age, maternal age, and fetal gender
