# Supplementary material for: Analysis of Biomarkers for Congenital Heart Disease Based on Maternal Amniotic Fluid Metabolomics
Source: Front Cardiovasc Med. 2021 Jun 7;8:671191. doi: 10.3389/fcvm.2021.671191 (PMC8215886; doi:10.3389/fcvm.2021.671191)
Supplement: Supplementary file 2 [file Table_2.DOCX]

Table S2. Differential metabolites of CHD in the validation set identified after adjustment for gestational week, maternal age, and fetal gender.

| Class | Metabolite | HMDB ID | Trend in CHD | OR(95% CI) | *P*-value^a^ |
| --- | --- | --- | --- | --- | --- |
| Amino Acids | Dimethylglycine | HMDB0000092 | ↑ | 1.84(1.06-3.19) | 0.029 |
|  | Proline | HMDB0000162 | ↑ | 2.20(1.20-4.03) | 0.011 |
|  | D-2-Hydroxyglutaric acid | HMDB0000606 | ↑ | 1.81(1.06-3.09) | 0.031 |
| Organic Acids | Uric acid | HMDB0000289 | ↑ | 3.24(1.62-6.48) | 0.001 |
|  | Oxoglutaric acid | HMDB0000208 | **↑** | 2.48(1.42-4.35) | 0.002 |
|  | Picolinic acid | HMDB0002243 | ↑ | 2.36(1.22-4.57) | 0.011 |
|  | Phenylpyruvic acid | HMDB0000205 | ↑ | 1.78(1.01-3.13) | 0.046 |
|  | Glutaric acid | HMDB0000661 | ↑ | 1.86(1.13-3.06) | 0.015 |
|  | Pyruvic acid | HMDB0000243 | ↑ | 2.12(1.22-3.69) | 0.008 |
|  | Malic acid | HMDB0000744 | ↑ | 2.01(1.16,3.50) | 0.014 |
| Carbohydrates | Gluconic acid | HMDB0000625 | ↑ | 1.82(1.07-3.09) | 0.027 |
|  | Galactonic acid | HMDB0000565 | ↑ | 2.09(1.11-3.92) | 0.022 |
|  | D-Maltose | HMDB0000163 | ↑ | 2.73(1.38-5.41) | 0.004 |
| Fatty Acids | Linoleic acid | HMDB0000673 | ↑ | 2.07(1.22-3.50) | 0.007 |
| Nucleotide | Uridine | HMDB0000296 | ↑ | 1.72(1.04-2.86) | 0.035 |
| Phenols | Dopamine | HMDB0000073 | ↑ | 1.92(1.10-3.34) | 0.022 |
| Alkylamines | Ethanolamine | HMDB0000149 | ↑ | 2.65(1.23-5.72) | 0.013 |
| NA | 3-hydroxypyridine | NA | ↑ | 1.63(1.02-2.60) | 0.043 |

HMDB,Human Metabolome Database; OR, odd ratio; NA, Not Available;

^a^ Logistic regression analysis adjusted for gestational age, maternal age, and fetal gender
